# Chronic urticaria merits serum vitamin D evaluation and supplementation; a randomized case control study

**DOI:** 10.1186/s40413-015-0066-z

**Published:** 2015-06-04

**Authors:** Roohi Rasool, Khalid Z Masoodi, Irfan A Shera, Qayser Yosuf, Imtiyaz A Bhat, Iqbal Qasim, Saniya Nissar, Zafar A Shah

**Affiliations:** Department of Immunology and Molecular Medicine, Sher-i- Kashmir Institute of Medical Sciences, Soura, Srinagar, J&K 190011 India; Division of Biotechnology, Sher-e-Kashmir University of Agricultural Sciences and Technology of Kashmir, Srinagar, J&K 190025 India

**Keywords:** 25 (OH) vitamin D, 5-D itch score, Chronic Urticaria, Visual analogue scale, IgE

## Abstract

**Background:**

Several studies suggest that Vitamin D (Vit-D_3_) supplementation reduces Chronic Urticaria (CU) symptoms.

**Objectives:**

Evaluation of serum 25-hydroxyvitamin-D (25 (OH)_2_D) level and assessment of therapeutic effect of VitD_3_in CU patients.

**Methods:**

192 subjects were stratified according to the baseline 25(OH)_2_D levels and subsequently randomized into three subgroups to receive Vit-D_3_ alone (VD) or antihistamine and systemic corticosteroid (H+S) or VitD_3_ with antihistamine and systemic corticosteroid (VD+H+S) for 6 weeks between July 2012 to Oct 2014. 130 healthy controls (HC) were followed without any intervention. The patients were evaluated for reduction in urticarial symptoms using visual analogue scale (VAS) and 5-D itch score.

**Results:**

Low serum levels of 25 (OH)_2_D was observed in 91% of CU patients and 64% of the healthy controls (P < 0.0001). VAS and 5-D Score in subgroups VD, H + S and VD + H + S decreased significantly from 6 · 7 ± 0 · 043, 6 · 6 ± 0 · 42 and 6 · 68 ± 0 · 40 at baseline to 5 · 2 ± 0 · 70 (P = 0 · 0088), 3 · 3 ± 0 · 50 (P < 0 · 0001) and1 · 86 ± 0 · 39 (P < 0 · 0001) after treatment and from 14 · 5 ± 0 · 72, 13 · 9 ± 0 · 77 and 13 · 9 ± 0 · 221 to 12 · 06 ± 1 · 10 (P = 0 · 0072), 8 · 1 ± 1 · 13 (P < 0 · 0001) and 5 · 01 ± 0 · 94 (P < 0 · 0001) respectively.

**Conclusions:**

CU patients have low serum 25(OH)_2_D levels and Vit-D_3_ supplementation in combination with antihistamine and systemic corticosteroid show elevated response in resolving the symptoms of CU. This study also warrants that each subject with CU should be screened for serum 25 (OH)_2_D levels before starting a treatment.

## Background

CU is defined as the presence of evanescent wheals which reoccur for greater than 6 weeks[1]. CU is allergic skin dermatosis, and despite extensive evaluation for underlying causes or triggers, the etiology remains unclear[2]. CU is a common skin disease that is rarely life threatening, however the high incidence and prevalence make it a paramount public health problem, which has protracted course and can result in significant morbidity[3]. Antihistamines and systemic steroids form the basis of treatment, but response is often incomplete [4]. Although second–line therapies, such as cyclosporine[5] and anti IgE therapy (omalizumab) [6], have been shown to be effective in randomized controlled trials, there are concerns about costs and adverse side effects associated with these therapies[7].

There are numerous studies suggesting potential role of Vit D in improving health outcomes, as far as allergic disorders are concerned. High prevalence of Vit D deficiency has also been reported in north Indian population[8]. Prevalence of atopic disorders in Vit D deficient patients when compared withVit D sufficient patients showed an increased risk of atopic dermatitis among those patients who were Vit D deficient. However, there was no significant difference in the risk of asthma or allergic rhinitis between Vit D deficient patients and Vit D sufficient patients[9]. Vit D deficiency has been shown to correlate with many food and environmental allergies in children[10]. Conversely, other studies have shown an association between high Vit D levels and the development of allergic rhinitis[11].

Although the molecular mechanism underlining the relation between CU and Vit D is still unknown, Vit D has been shown to play a considerablerole in the immune responses generated by antigen-presenting cellsthat has been recognized with the discovery of Vit D receptors(VDRs) on these cells[12]. VDRs have been present on a variety of cells, including keratinocytes and numerous cells of immune system (e.g. T cells, B cells, neutrophils, macrophages, and dendritic cells) [13]. The scope of biological role of the action of Vit D through VDRs helped clarify the link between Vit D and immune functions[14]. Also, Vit D affects innate and adaptive immunity through the stimulation of Toll-like receptors, increasing pro-inflammatory cytokine production, and possibly enhancing T helper type 2 responses, thereby defining the possible association of Vit D to allergic disorders. Many reports suggest a possible influence of Vit D on prevalence of allergic diseases even though results are still conflicting [15].

Though there have beenfewer studies that have evaluated the potential link between Vit D and urticaria[16,17], we sought to determine whether a relationship between Vit D and CU exists.

Though there have been mixed reviews about the use of Vit D as treatment in CU,Vit D has been successfully used as a treatment modality in psoriasis and lupus vulgaris[18,19]. Skin lesions were resolved when Vit D insufficiency was treated with oral supplementation of Vit D[20]. Likewise no cutaneous consequences of low Vit D are described in Holick’s extensive reviews of the health implications of Vit D deficiency and inadequacy[21]. Recently it has been determined that a high dose (4,000 IU/day) of Vit D3 is more beneficial than a low dose (600 IU/day) which can be used as an add-on therapy in CU patients. High dose of Vit D3 can be considered as safe in patients with CU[22].

In light of several conflicting hypotheses, we conducted a randomized controlled study to investigate the efficacy and therapeutic benefits of different treatment regimens with and without Vit D_3_supplementation inCU patients.

## Methods

### Study population

Patients aged 18 to 60 years with a clinical diagnosis of moderate-to-severe CSU were evaluated in the present investigation. The criteria for CSU severity was assessed according to the classified consensus guidelines from the European Academy of Allergology and Clinical Immunology [EAACI] and the World Allergy Organization [WAO] [23,24].

### Evaluation of the CU symptoms

The 5-D itch scale is a multidimensional questionnaire designed to be useful as an outcome measure in clinical trials[25]. The five dimensions are;degree, duration, direction, disability and distribution of pruritus per 24 hours. The scores of each of the five domains are calculated separately and then summed together to obtain a total 5-D score. 5-D scores can potentially range between 5 (no pruritus) and 25 (most severe pruritus). Randomization was performed by using a validated system that automated the random assignment of the treatments to randomization numbers. The present investigation consisted of a pre-screening visit, a 4 week screening period and a 6 week treatment period and 4 weeks follow up. At the prescreening visit, informed consent was obtained, and patients were assessed for eligibility. VAS was used as a method of pruritis assessment in CSU patients[26].

### Inclusion criteria

The patients with persistent symptoms for more than 6 weeks at screening, body weight between 25 and 100 kg, a total serum IgE level ranging between 30 IU/mL to700 IU/mL, and a weekly 5-D score of 10 or greater and visual activity score (VAS) of 5 or greater at the end of screening were eligible for enrollment in the study. Serum 25 (OH)_2_D levels <10 ng/ml reflect severe Vit D deficiency, levels between 10–20 ng/ml depicted as deficient, levels 20–30 ng/ml were described as insufficient and 25 (OH)_2_D levels of > 30 ng/ml were defined as sufficient levels [27,28].

### Exclusion criteria

Patients having lesions that last more than 24 hours, and having physical urticaria syndromes were not included in this study. Patients were excluded if they had received antihistamines, systemic steroids or immunosuppressive therapy during the preceding 3 months. Patients that were having morbidity like diabetes, hypertension, dermatitis herpetiformis and mastocytoma and any form of kidney disease were also excluded. Patients having hypercalcemia or any other calcium disorders were also excluded from the study. Patients with urticaria caused by infection, food allergy and drug allergy were also excluded.

### Baseline investigations

Baseline investigations in all patients included a routine laboratory tests like full blood count, erythrocyte sedimentation rate, urine analysis, serum glucose, hepatic functions and creatinine. Patients were also tested for hepatic serology, thyroid function test, antinuclear and anti-thyroid microsomal antibodies. Chest X-ray and abdominal ultrasound were also performed on the patients. The patients also underwent the detection for *Helicobacter pylori*. Autologous serum skin testing (ASST), skin prick test (SPT) were also performed on all patients.

### Safety assessment

Safety assessment included the monitoring and recording the severity of adverse events, along with evaluation of their duration and relationship to the study. In addition, routine urine analysis, regular monitoring of hematology and blood chemistry results and assessment of vital signs and body weight were performed[24]. Patients were also monitored for calcium levels at week 2, 4 and 6.

### Study design and treatment groups

Measurement properties were analyzed using data from randomized, parallel- group study of orally administered Vit D as add-on therapy for the treatment of CSU. 505 patients and 210 normal matched healthy controls were assessed for eligibility and finallynarrowed down to 192 and 130 respectively from the outpatient department of Allergy/Immunology ofthe Sher-i-Kashmir Institute of Medical Science,Srinagar,in the period between July 2012-Oct 2014. The study was approved by the ethics committee of the institutionand was conducted in accordance with the International Conference on Harmonization Guidelines for Good Clinical Practice, the ethical principles embodied in the Declaration of Helsinki (1989), and applicable local regulations. Written informed consent was obtained from all patients before their participation in the study.

### Experimental design

**Figure 1:**
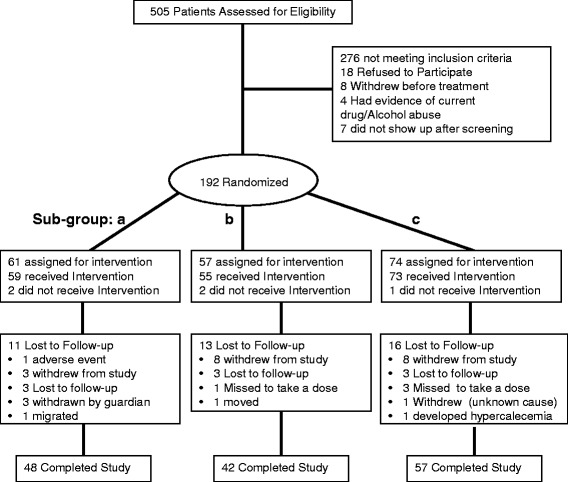
**Enrollment and outcome.** 505 patients were enrolled for eligibility out of which only 192 fulfilled the eligibility criteria. 276 patients did not fulfill the criteria, 18 patients refused to participate, 8 withdrew before treatment, 4 patients having evidence of current drug/alcohol abuse were not recruited for the study and 7 did not show up after screening. 192 patients fulfilling the eligibility criteria were randomized into groups **a**, **b** and **c**. 48 patients in group **a**, 42 in group **b** and 57 in group **c** completed the study. The rest withdrew from study, had adverse events, missed to take the dose, were withdrawn by the guardian or were lost to follow-up.

**Figure 2:**
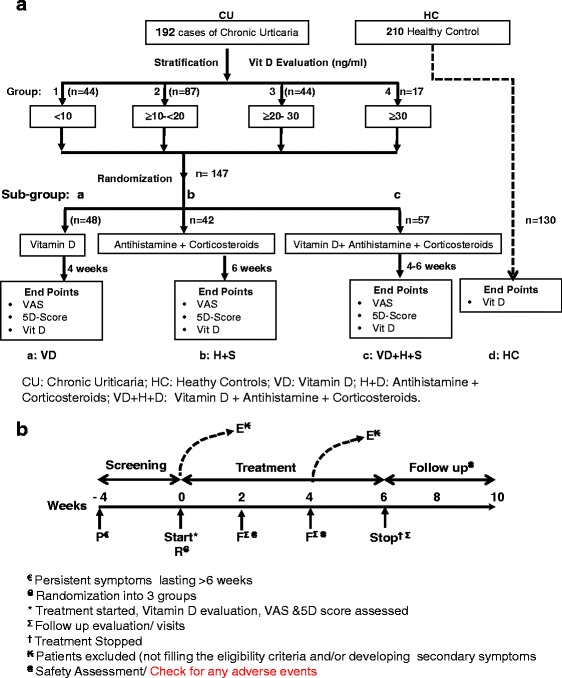
**Experimental protocols. a**. Vitamin D evaluation and treatment regimen of Chronic Urticaria patients. Patients were stratified into 4 groups depending upon VitD levels. Group 1, 2, 3 and 4 consisted of patients with serum 25(OH)_2_D levels <10ng/ml, ≥ 10 ng/ml to < 20 ng/ml, ≥ 20 to 30 ng/ml and >30ng/ml respectively. All the groups were further randomized into 3 treatment protocol regimens, a, b and c. 48 Patients in Protocol **a** received Vitamin D (VD) for 4 weeks, 42 patients in group **b** received antihistamines and corticosteroids (H+S) for 6 weeks and 57 patients in group **c** received a combinatorial therapy of vitamin (4 weeks) + antihistamines+ corticosteroids (VD+H+S) for 6 weeks. 130 healthy controls (HC) in group **d** were followed as such without any intervention. Patients were tested for Vit D, VAS and 5D score at the baseline and after 6 weeks of treatment. **b**. Outline of experiment starting from recruitment to attaining the end point. Participants were screened for eligibility starting 4 weeks before the start of the experiment (Screening). The patients that do not fill the eligibility criteria and/or developed secondary disorders during the treatment were excluded from this study (E^₭^). Follow up visit for safety assessment was at 2,4,6,8 and 10 weeks after the start of the study (F^₴^). The treatment lasted for 6 weeks with follow up and safety assessment for another 4 weeks. The sample size calculations was chosen in a way that the test for the primary end point, the mean weekly VAS and 5-D score, would have 85% power by using a 2-sided 5% significance level of the treatment effect (i.e., the difference between the treatment groups) was at least as large as its SD (standardized effect size, >1).

Subjects were initially divided into two groups, chronic urticaria (CU) and Healthy control (HC). 505 patients were initially screened for the study out of which 63.9% did not fulfill the eligibility criteria or refused to participate or had evidence of drug and/or alcohol abuse (Figure 1). Baseline serum Vit D was evaluated as 25-hydroxyvitamin D (25(OH)_2_D) using enzyme immuno assay in all the subjects. 192 CU patients fulfilling the eligibility criteria,were stratified into 4 Groups. Patients with serum levels <10 ng/ml reflecting severe vitamin deficiency were included in group 1. Patients having 10–20 ng/ml of serum 25 (OH)_2_ D depicted deficient levels and were included into group 2. The patients in which the level of 25 (OH)_2_ D ranged between 20–30 ng/ml were described as insufficient and comprised group 3 and the patients where 25(OH)_2_ D levels greater than 30 were defined as sufficient and comprised group 4. The four groups were then randomized and treated using three different protocols; a, b and c (Figure 2a).

The four groups were randomized into 3 subgroups a, b and c. Out of these, 48 in subgroup a, 42 in subgroup b and 57 in subgroup c completed the study (Figure 2a). The rest were lost to follow-up, had adverse events, were withdrawn by guardian, missed a dose or developed hypercalcemia (Figure 2b). The experimental design is outlined in Figure 2a & b. All subjects satisfying entry criteria were randomized to receive three different treatment regimens.

In subgroup **a** (VD), 48patients received oral Vit D_3_ (cholecalciferol) sachets at a dose of 60,000 IU per week for 4 weeks.In subgroup **b** (H + S), 42 patients received, antihistamine (Hydroxyzine 25 mg) and corticosteroids (Deflazacort 6 mg)PO daily for 6 weeks. In subgroup** c** (VD + H + S), 57 patients received combinatorial therapy with oral VitD_3_ sachets at a dose of 60,000 IU per week for 4 weeks, antihistamine PO, corticosteroids PO daily for 6 weeks. Healthy control group (n = 130) was followed as such without any intervention for 6 weeks as described in protocol d (HC) (Figure 2a). The patients were followed up to 10 weeks for safety assessment from the start of the treatment regimen to see if the patients had recurrence of symptoms or any other off target effects or any adverse after effects of the treatment.

### Monitoring and study end points

Follow up visit was done at two, four and six weeks to assess the treatment efficacy for CU and any toxic effects of Vit D. At each visit, any adverse effects of treatment regimens were recorded. The mean reduction in 5-D score and VAS after 6 weeks of treatment compared with the baseline score signifies therapeutic response of treatment regimens in CU.

Blood was collected at prescreening, at baseline, at week two, week four and week six after treatment from each subject of Subgroup a, b, c and d, to evaluate 25(OH)_2_VitD levels and to ensure none exceeded moderate normal values.

### Statistical analysis

The sample size was chosen to ensure 85% power for the primary end point, the mean weekly VAS and 5-D score, by using a 2-sided 5% significance level to detect a treatment effect at least as large as its SD (standardized effect size, >1). Data was expressed as Mean ± standard error mean (SEM). The difference in the mean values of Vit D in both the cases and control groups were analyzed by paired *t*-test to calculate the difference within groups and Bonferroni independent samples *t*-test was used to calculate the level of difference between groups. The primary end point (VAS and 5D-Score) was evaluated by using an analysis of covariance model with the factors of treatment and center and with the baseline score as the covariate. For the treatment contrast among the three subgroups a, b and c, a 95% CI and a 2-sided P value were calculated. Mean score (VAS, 5-D) changes within subgroup a,b and c were compared, using paired *t*-test (keeping normality of the observation into considerations). Intergroup comparison at different time-points was done using analysis of variance test (ANOVA). A *P* < 0 · 05 was taken to be statistically significant. Statistical analysis was performed using GraphPad Prism version 6.00 for Windows (GraphPad Software, San Diego California USA.

## Results

### Demographics and vitamin D levels

A total of 192CU patients were enrolled in the present investigation out of which only 147 were considered for the final evaluation (Figure 2). There was no significant difference in age of CU Patients (mean years ± SD; 42 · 83 ± 8 · 52) vs controls (45 · 12 ± 7 · 65)and patients were predominantly female (67 · 7%). 18 · 9% of patients were smokers and 25 · 4% of the healthy control had been smoking over a year. Mean ± SEM Vit D was 17 · 87 ± 1 · 22 ng/ml for subjects versus 27 · 65 ± 1 · 65 ng/ml for controls.

**Figure 3:**
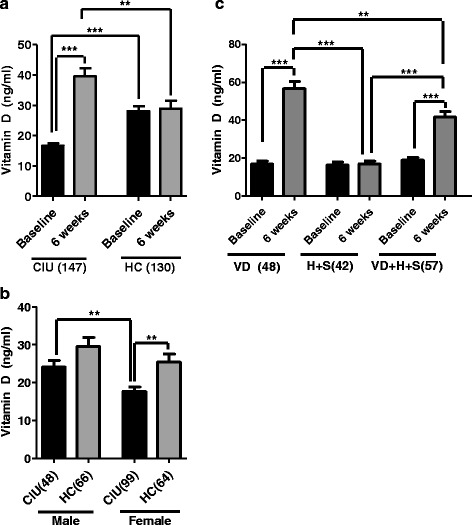
**Vit D status in CU patients and healthy controls at baseline and after 6 weeks of treatment. a**. Quantification of serum 25(OH)_2_D levels in CU patients and healthy controls at baseline and after 6 weeks of treatment. Data represents 147 CU patients and 130 healthy controls. Error bars represent SEM. Paired *t*-test was used to calculate the difference within groups and Bonferroni independent samples *t*-test was used to calculate the level of difference between groups. **P < 0.01and ***P < 0.0001. **b**. Comparative analysis of baselineVit D levels in male and female CU patients and healthy controls. Error bars represent SEM. Number of patients in each group is represented in parentheses. **P < 0.01. **c**. Serum 25(OH)_2_D levels in CU patients undergoing treatment under three regimens at baseline and after 6 weeks of treatment. Patients were treated with protocols VD, H + S and VD + H + Sand serum Vit D was measured at baseline and after 6 weeks. Paired *t*-test was used to calculate the difference within groups. Error bars represent SEM. **P < 0.01; ***P < 0.0001.

Vit D deficiency or insufficiency was seen in 91 · 3% of the subjects with CU and 63 · 84% of the healthy controls. Vit D levels were significantly lower (P <0 · 0001) in subjects with CU compared with controls (Figure 3a). The proportion of all subjects with Vit D deficiencywas not significantly different between the cases and controls in men (Figure 3b). However, females with CU had predominantly lower levels of Vit D (17 · 7 ± 1 · 12 ng/ml) compared to controls (25 · 44 ± 2 · 24 ng/ml) (**P = 0 · 0018) (Figure 3b). Also women showed significantly lower levels of Vit D in comparison to men with CU (P = 0 · 0019) (Figure 3b) [27,28]. Autologous serum skin test (ASST) results were positive in 58 · 2%of the patients with CU.

### Treatment response

#### Effect of vitamin D supplementation on serum 25(OH)_2_D levels

At enrollment, there was no significant difference in total baseline Vit D levels between subgroups a, b and c (Figure 3c). After 6 weeks of treatment, subgroup **a** undergoing Vit D supplementation alone showed a 3.3 fold increase in serum 25(OH)_2_D from 16.98 ± 1.43 ng/ml to 56.74 ± 3.76 ng/ml (Mean ± SEM) with ***P < 0 · 0001 (Figure 3c). There was no significant change in 25(OH)_2_Dfrom 17.04 ± 1.54 ng/ml to 16.44 ± 1.50 ng/ml in subgroup **b **(Figure 3c). In subgroup **c** (VD + H + S), there was 2.2 fold increase in total serum 25(OH)_2_ D levels from 18.95 ± 1.42 ng/ml to41.73 ± 2.85 ng/ml (***P < 0 · 0001) (Figure 3c).

**Figure 4:**
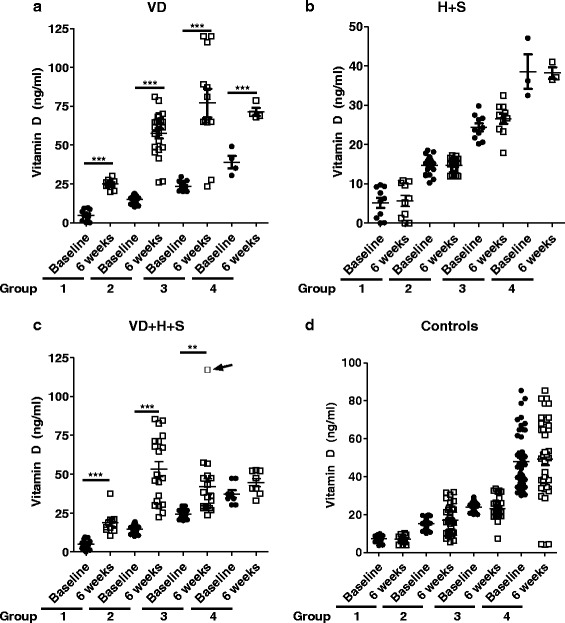
**Serum 25(OH)**_**2**_ **D levels in CU patients undergoing treatment under three treatment regimens at baseline and after 6 weeks of treatment. a**. Patients were treated with protocols VD and serum Vit D was measured at baseline and after 6 weeks. Paired *t*-test was used to calculate the difference within groups. Error bars represent SEM. ****P < 0.0001. **b**. Patients were treated with protocols H + S and serum Vit D was measured at baseline and after 6 weeks of treatment. **c**. Patients were treated with protocols VD + H + S and serum Vit D was measured at baseline and after 6 weeks of treatment. The arrow depicts the patient that developed hypercalcemia. **d**. Vit D levels in controls subjects. Paired *t*-test was used to calculate the difference within groups. Error bars represent SEM. **P < 0.01; ****P < 0.0001. Vitamin D (VD), Hydroxizine (H) and Deflazacort (S).

Subgroup **a** showed a significant increase in the serum 25 (OH)_2_D levels across all the four groups 1–4 (Figure 4a). Group 1,2, 3 and 4 had a baseline 25 (OH)_2_ D levels of (Mean ± SEM ng/ml) 4.89 ± 1.2, 14.96 ± 0.57, 23.46 ± 0.93 and 38.95 ± 4.03 which increased to 24.91 ± 0.97 (P < 0.0001), 57.49 ± 3.07 (P < 0.0001), 77.02 ± 9.29 (P = 0.0001) and 71.39 ± 2.44 (P = 0.0006) respectively (Figure 4a). In subgroup **a** the patients having critically low levels of serum 25 (OH)_2_D before treatment, showed a 5 fold increase after 6 weeks of treatment. Group 2 having deficient levels of serum 25 (OH)_2_D showed almost 4 fold increase with serum 25 (OH)_2_D levels attaining sufficiency. Similarly group 3 having insufficient levels of serum 25 (OH)_2_D showed 3 fold increase and attained sufficiency. The patients already having sufficient levels of serum 25 (OH)_2_D also showed less than 2 fold increase.

There was no significant change in all the four groups 1–4 in subgroup **b **(Mean ± SEM ng/ml) 5.642 ± 1.4, 14.69 ± 0.43, 26.54 ± 1.3 and38.21 ± 1.42) when compared to baseline 25(OH)_2_ D serum levels (5.141 ± 1.23, 14.71 ± 0.55, 24.36 ± 1.00 and 38.58 ± 4.38) respectively (Figure 4b). In subgroup **b** the patients did not show any significant change in any of the 4 groups as expected.

Subgroup **c** also showed a significant increase in serum 25(OH)_2_D levels in group 1, 2 and 3 (Figure 4c). The serum 25(OH)_2_D levels increased from baseline 4.987 ± 0.97, 14.53 ± 0.59 and 24.28 ± 0.66 to 18.69 ± 2.12 (P < 0.0001), 53.10 ± 4.81 (P < 0.0001) and 41.92 ± 5.06 (P = 0.0043) respectively. However, group 4 did not show any significant increases from baseline 37.22 ± 2.38 to 44.56 ± 2.67 at week 6 (Figure 4c). In subgroup **c** the patients having critically low levels of serum 25 (OH)_2_D before treatment, showed a 3.7 fold increase after 6 weeks of treatment. Group 2 having deficient levels of serum 25 (OH)_2_D showed 3.6 fold increase with serum 25 (OH)_2_D levels attaining sufficiency. Group 3 having insufficient levels of serum 25 (OH)_2_D showed less than 2 fold increase and attained sufficiency. However the change was not significant when compared to group 3 of subgroup **a**. One patient in group 3 subgroup c showed an elevated vitamin D after 4 weeks of treatment with combinatorial therapy. The patient when evaluated for for basic biochemistry showed an elevated serum calcium levels. After stopping the treatment the patient retained normal calcium levels in less than 2 days. In this time the patent was monitored continuously.

Healthy Control group did not show any significant change in all the four groups with serum 25(OH)_2_ D levels at baseline (7.310 ± 0.52, 15.26 ± 0.47, 23.98 ± 0.46 and 47.78 ± 2.23) to (5.899 ± 0.28, 16.96 ± 1.26, 23.15 ± 0.95 and 49.18 ± 2.97) (Figure 4d). All the groups of control subgroup **d** did not show any change in serum 25 (OH)_2_D levels as expected.

Moreover, no adverse effects were observed in the study subjects after two, four and six weeks of treatment except for one patient that developed hypercalcemia in subgroup c (VD + H + S) and was withdrawn from the study.

#### VAS score

**Figure 5:**
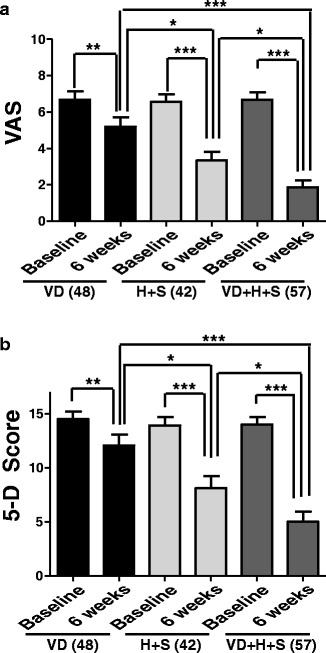
**Effect of Vitamin D supplementation in different therapy protocols for CU: a**. Effect of Vitamin D (VD), antihistamines(H) and corticosteroids (S) on VAS of CU patients. Patients were treated with VD alone, H + S and VD + H + S for 6 weeks. VAS was calculated at the start of the study (baseline) and after 6 weeks of treatment. Values are presented as mean ± SEM. *, P < 0.05; **, P < 0.01; ***,P < 0.0001. Number of patients in each group is represented in parentheses. **b**. Effect of Vitamin D (VD), antihistamines(H) and corticosteroids (S) on 5-D score of CU patients. Patients were treated with VD alone, H + S and VD + H + S for 6 weeks. 5-D score was calculated at the start of the study (baseline) and after 6 weeks of treatment. Number of patients in each group is represented in parentheses. Values are presented as mean ± SEM. *, P < 0.05; **, P < 0.01; ***,P < 0.0001. The primary end point (VAS and 5D-Score) was evaluated by using an analysis of covariance model with the factors of treatment and center and with the baseline score as the covariate. For the treatment contrast between the three groups a, b and c, a 95% CI and a 2-sided P value were calculated. Mean score (VAS, 5-D) changes within group VD and group H + S and group VD + H + S were compared, using paired *t*-test (keeping normality of the observation into considerations). Intergroup comparison at different time-points was done using analysis of variance test (ANOVA). A *P* < 0 · 05 was taken to be statistically significant.

The mean VAS score in subgroup**a**decreased significantly from a baseline score of 6 · 7 ± 0 · 043 to the score of 5 · 2 ± 0 · 70 with *P = 0 · 0088(Figure 5a). The mean VAS score of subgroup **b** was significantly reduced to half from 6 · 6 ± 0 · 42 at baseline to 3 · 3 ± 0 · 50 with ***P < 0 · 0001(Figure 5a). Similarly the mean VAS score in subgroup **c**was reduced significantly by 3 · 6 fold from baseline 6 · 68 ± 0 · 40 to 1 · 86 ± 0 · 39 with***P < 0 · 0001(Figure 5a). Comparing inter group VAS score there was a significant differencein subgroup **b** compared to subgroup **a** (*P = 0 · 0106) at week 6. Subgroup **a** also showed a significant difference in VAS when compared to the subgroup **c**(***P < 0 · 0001), VAS score in subgroup **c**also showed a significant differencein comparison to subgroup **b** with *P = 0 · 0203(Figure 5a).

### 5-D itch score

The mean 5-D itch score in subgroup **a**decreased significantly from a baseline score of 14 · 5 ± 0 · 72 to the score of 12 · 06 ± 1 · 10 with **P = 0 · 0072(Figure 5b). The mean 5-D score of subgroup **b** was significantly reduced to half from 13 · 9 ± 0 · 77 at baseline to 8 · 1 ± 1 · 13with ***P < 0 · 0001 (Figure 5b). Similarly the mean 5-D score in subgroup **c** was reduced significantly by 2 · 8 fold from 13 · 9 ± 0 · 68 to 5 · 01 ± 0 · 94 with ***P < 0 · 0001(Figure 5b).

Comparing inter subgroup 5-D score there was a significant difference in subgroup **b** compared to subgroup **a** (*P = 0 · 0116). Subgroup **a** also showed a significant difference in 5-D score when compared to the group **c** (***P < 0 · 0001). 5-D score in subgroup **c** also showed a significant difference in comparison to subgroup **b** with *P = 0 · 0382 (Figure 5b).

## Discussion

CU encompasses a heterogeneous group of disorders with diverse underlying etiologies. However, upto 40% of patients with CU are autoimmune in nature, demonstrate auto antibodies against either high-affinity IgE receptors (FcƐRI subunit) or IgE antibody on mast cell surfaces[3]. Degranulation of mast cells with release of histamine is central to the pathogenesis of urticaria[29]. H1 antagonists are firmly established as first line therapy for CU with adjunctive support from medications of other classes, such as antileukotrienes, immunosuppressive and anti-inflammatory agents (including steroids and cyclosporine) [30],[31]. Vit D has anti-inflammatory properties, as observed by the 1, 25 (OH) 2 D mediated reduction of dendritic cell (DC) maturation [32]. Furthermore, Vit D contributes to the conversion of CD4+ T cells to T regulatory cells, which have been shown to play a role in the suppression of pro-allergic mechanisms[33].

Vit D deficiency is presently a major health problem in both adults and children across the globe and highly prevalent in Indian sub-continent [8,34,35]. The study population was predominately ethnic, consistent with the region and had Vit D_3_ insufficiency or deficiency. We devised a randomized study to evaluate whether correcting hypovitaminosis D had any therapeutic effectiveness on CU. Theprimary objective of our study was to ascertain a potential link between Vit D_3_ deficiency and CU and the effect of VitD_3_ supplementation in repressing the symptoms of CU. A high percent (91.3%) of CU patients had insufficient or deficient 25 (OH)_2_D levels against 63.8% of the healthy control participants, suggesting a possible role of Vit D_3_deficiency in the pathogenesis and exacerbation of CU. These findings provide a detailed investigation of the beneficial effect of Vit D_3_ supplementation in CU patients. The present investigation further support and are consistent with the previous studythat showed CU patients have significantly low levels of 25 (OH)_2_D [16] and Vit D_3_ supplementation decreases the CU symptoms and increases the quality of life of the patients diagnosed with CU [22].

A high dose of Vit D_3_(60,000 IU/week for4weeks) was used in the present study because there is high prevalence of Vit D_3_ deficiency and/or insufficiency in the Kashmiri population[8] and beneficial effect of high dose of Vit D_3_ over low dose in the treatment and recovery of patients with CU has been reported recently[22]. The notion behind using Vit D_3_ instead of Vit D_2_ in the present investigation was that Vit D3 is more potent for raising and maintaining steady-state 25(OH)_2_D levels than VitD2 [34,36].

CU is intractable skin disease, antihistamines and systemic steroids form the basis of treatment, but response is often incomplete [4]. An association of pruritus, rash, and urticaria/ angioedema with low 25(OH)_2_D <32 ng/mlhas also been reported,andresolution of symptoms following Vit D_3_ replacement was observed in 70% of those patients with cutaneous symptoms and concurrent Vit D_3_deficiency [20], however no detailed extensive study has beenreported so far on the efficacy of Vit D_3_ supplementation in resolving the symptoms of CU. In the present investigation we observed the patients that receivedVit D_3_as monotherapy showed improvement in the CU symptoms. The patients that received combinatorial therapy showed better improvement of symptoms and quality of life than the patients who received only standard therapeutic regimen [37].

Although the patients receiving Vit D_3_alone and the patients receiving standard treatment with Vit D_3_ supplementation received equal amount of VitD_3_, there was still a significant difference in the serum 25 (OH)_2_D levels between these twosubgroups, with subgroup **a** showing significantly higher levels of VitD_3_ then subgroup **c**. This difference can be attributed to the effect of systemic corticosteroids on the 25 (OH)_2_D levels [38].

Althoughthe course of CU is often quite variable and protracted;a short follow-up of just 6 week was enough to show a significant effect on Vit D, VAS and 5-D score. Furthermore, the patients with CU who exhibit incomplete response to drug therapy continue to have symptoms, although there might be a reduction in their symptoms. For this reason, the VAS and 5D-itch scoresare likely more reliable indicators of treatment response in short duration studies.

In view of present study, role of Vit D_3_alone as treatment for CU seems conclusive, although combinatorial therapy proved more effective form of treatment in patients with CU. The exact underlying mechanism needs still to be investigated even though the possible reason might be adjunctive action from different classes of drugs in diverse pathogenesis of CU.

## Conclusions

Our study showed Vit D_3_ levels are significantly reduced in subjects with CU compared with controls. The study provides a rationale for the selection of Vit D supplementation as “add-on” therapy for the treatment of CU with patients showing a greater resolution of CU symptoms.

## Abbreviations

CU: Chronic Urticaria
ASST: Autologous serum skin testing
VAS: Visual analogue scale
VDRs: Vitamin D receptors
VD: Vitamin D
